# Draft genome sequence data of *Cercospora kikuchii*, a causal agent of Cercospora leaf blight and purple seed stain of soybeans

**DOI:** 10.1016/j.dib.2019.104693

**Published:** 2019-10-21

**Authors:** Francisco J. Sautua, Sergio A. Gonzalez, Vinson P. Doyle, Marcelo F. Berretta, Manuela Gordó, Mercedes M. Scandiani, Maximo L. Rivarola, Paula Fernandez, Marcelo A. Carmona

**Affiliations:** a Universidad de Buenos Aires, Facultad de Agronomía, Cátedra de Fitopatología, Buenos Aires, Argentina; b Instituto de Biotecnología, IABIMO, CICVyA, Instituto Nacional de Tecnología Agropecuaria, Hurlingham, Buenos Aires, Argentina; c Department of Plant Pathology and Crop Physiology, LSU AgCenter, Baton Rouge, LA, 70803, USA; d Consejo Nacional de Investigaciones Científicas y Técnicas (CONICET), Buenos Aires, Argentina; e Instituto de Microbiología y Zoología Agrícola (IMyZA), Centro de Investigaciones en Ciencias Veterinarias y Agronómicas (CICVyA), Instituto Nacional de Tecnología Agropecuaria (INTA), Buenos Aires, Argentina; f Laboratorio Agrícola Río Paraná, San Pedro, Argentina; g Universidad Nacional de Rosario, Facultad de Ciencias Bioquímicas y Farmacéuticas, Centro de Referencia de Micología (CEREMIC), Rosario, Argentina; h Universidad Nacional de San Martín, Instituto de Investigaciones Biotecnológicas, Argentina

**Keywords:** *Cercospora kikuchii*, Draft genome, Next generation sequencing (NGS), Cercospora leaf blight (CLB), Purple seed stain (PSS), Agriculture, Bioinformatics, Fungal pathogens

## Abstract

*Cercospora kikuchii* (Tak. Matsumoto & Tomoy.) M.W. Gardner 1927 is an ascomycete fungal pathogen that causes Cercospora leaf blight and purple seed stain on soybean. Here, we report the first draft genome sequence and assembly of this pathogen. The *C. kikuchii* strain ARG_18_001 was isolated from soybean purple seed collected from San Pedro, Buenos Aires, Argentina, during the 2018 harvest. The genome was sequenced using a 2 × 150 bp paired-end method by Illumina NovaSeq 6000. The *C. kikuchii* protein-coding genes were predicted using FunGAP (Fungal Genome Annotation Pipeline). The draft genome assembly was 33.1 Mb in size with a GC-content of 53%. The gene prediction resulted in 14,856 gene models/14,721 protein coding genes. Genomic data of *C. kikuchii* presented here will be a useful resource for future studies of this pathosystem. The data can be accessed at GenBank under the accession number VTAY00000000 https://www.ncbi.nlm.nih.gov/nuccore/VTAY00000000.

**Table:** Specifications table

Subject	Biology
Specific subject area	Bioinformatics (Genomics)
Type of data	Raw sequencing reads, draft genome assembly, gene prediction and phylogenetic position of *C. kikuchii* strain ARG_18_001
How data were acquired	Whole genome sequencing was performed using an Illumina NovaSeq 6000 sequencing system
Data format	Raw sequencing reads, draft genome assembly and gene prediction
Parameters for data collection	Reads were filtered and merged with Trimmomatic (v 0.39) and FLASH (v 1.2.11). The genome was assembled with Celera Assembler (v 8.3) and Spades (v 3.11.1). Gene prediction was performed with FunGAP (v 1.0.1), tRNAscan-SE (v 2.0.3), rnammer (v 1.2) and mfannot (v 1.35). Protein-coding gene annotation was performed with hmmsearch (v 3.1b2), ncbi-blast (v 2.2.25+) and Blast2GO (v 2.5) using the ragp R package (v 0.3.0.0001). RepeatMasker (v 4.0.9) was used to identify and filter repetitive regions.
Description of data collection	Strain ARG_18_001 was isolated from soybean seeds of variety DM62R63 sampled during the 2018 harvest that exhibited symptoms of purple seed stain.
Data source location	Samples were originally collected from Gobernador Castro, San Pedro, Buenos Aires, Argentina (33°39′26.37″S, 59°49′36.00″O)
Data accessibility	This Whole Genome Shotgun project has been deposited at DDBJ/ENA/GenBank under the accession VTAY00000000 https://www.ncbi.nlm.nih.gov/nuccore/VTAY00000000. The version described in this paper is version VTAY00000000.1 https://www.ncbi.nlm.nih.gov/nuccore/VTAY00000000.

**Table:** 

**Value of the Data** • The first draft genome of *Cercospora kikuchii* ARG_18_001. • *C. kikuchii* is an important pathogen of soybean, but the biology of this fungus is poorly understood. • Genomic data presented here will be a useful resource for the study of this pathosystem. • This draft genome will help in the search for genetic resistance in soybean lines

## 1 Data

**Fig. 1:**
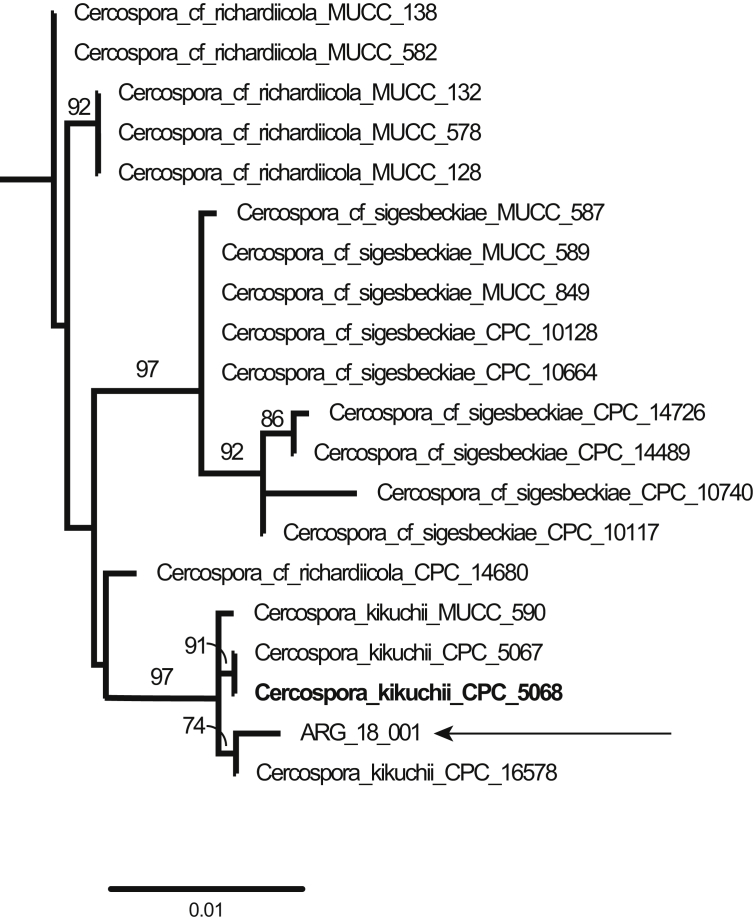
Subtree from a maximum-likelihood phylogenetic analysis of *Cercospora* species. The complete phylogeny was inferred in RAxML assuming the GTRGAMMA model by integrating data sliced from the genome of ARG_18_001 with the following seven loci from 379 other isolates in Groenewald et al. (2013) and Bakhshi et al. (2018): *actA*, *cmdA*, *nrITS*, *gapdh*, *histone 3*, *tef1-alpha*, and *tub2*. The subtree that includes ARG_18_001 was pruned from the rest of the tree for ease of reference. Branches are labeled with bootstrap support values ≥ 70%. Bold font indicates the placement of the ex-type of *C. kikuchii*. Arrow indicates the placement of the isolate for which the genome was sequenced. The scale bar indicates the estimated number of substitutions per site.

We present the draft genome assembly and gene prediction of the fungus *C. kikuchii*, causal agent of Cercospora leaf blight (CLB) and purple seed stain (PSS) of soybean. Recently, multi-locus phylogenetic studies confirmed that CLB and PSS is a disease complex caused by several *Cercospora* species. Phylogenetic analyses of cercosporoid fungi isolated from infected soybean in Argentina, Brazil and the USA determined that the species *C. kikuchii*, *C.* cf. *flagellaris* and *C.* cf. *sigesbeckiae* are causal agents of these diseases [1,2]. More recently, *C.* cf. *nicotianae* isolated from soybean leaves in Bolivia has been identified as a species in association with CLB [3]. A maximum-likelihood phylogenetic tree of *Cercospora* species was inferred in RAxML using seven nuclear loci, with data from isolate ARG_18_001 sliced from the genome assembly. The strain ARG_18_001 nested within the clade that includes other isolates of *C. kikuchii*, including the ex-type, with 97% bootstrap support (Fig. 1).

**Fig. 2:**
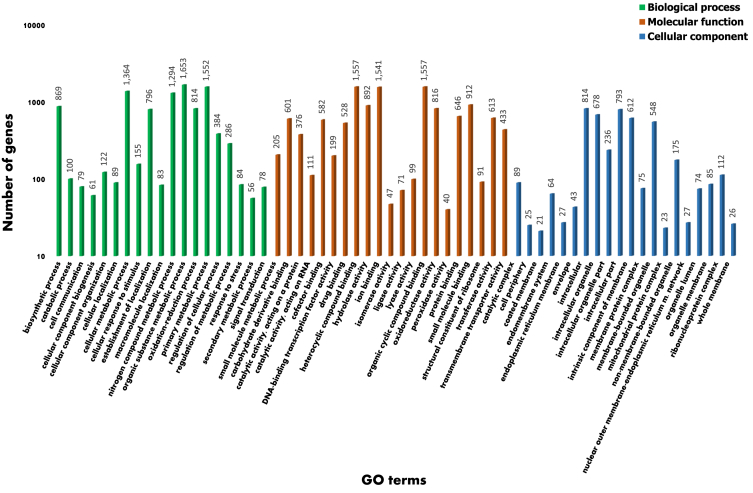
Histogram representing the gene ontology distribution of the annotated *Cercospora kikuchii* ARG_18_001 genes. The functionally annotated genes were assigned to three main GO categories: Biological Process (BP), Molecular Function (MF) and Cellular Component (CC).

**Fig. 3:**
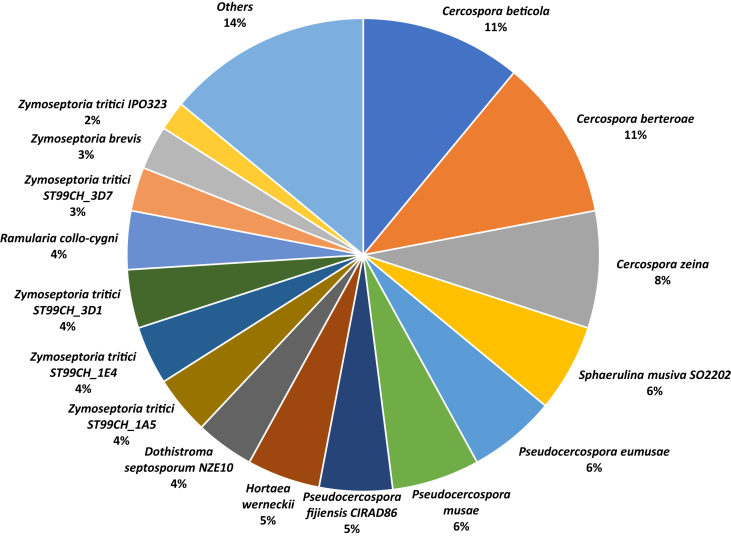
Pie chart denoting the species distribution based on the top BLAST hit of the *Cercospora kikuchii* ARG_18_001 genes queried against the nr database with an E-value cut-off of 1E-10. The category “Others” includes species with less than 1% representation.

A total of 33,107,531 reads were assembled de novo, resulting in 136 scaffolds of at least 500 bp with the largest scaffold 3,211,885 bp and an N50 value of 898,622 bp. The mean coverage of the total assembly was 196.72-fold. The G + C content was 53.04%. The gene prediction resulted in 14,856 gene models with 14,721 protein coding genes and 135 non coding RNAs, including the mitochondrial genome (Table 1). The distribution of protein annotations are summarized in Table 2, and Table 3 provides the summary statistics of the identified repetitive elements. The distribution of functional gene ontology (GO) terms from the annotated *C. kikuchii* ARG_18_001 genes are illustrated in Fig. 2. The distribution of species from the top BLAST hit of the predicted protein coding genes is shown in Fig. 3.

**Table 1:** Genome features of *C. kikuchii* strain ARG_18_001.

Features	*C. kikuchii* ARG_18_001
Assembled length	33,197,932
Scaffold length (≥ 50,000 bp)	32,541,287
Number of scaffolds (>500 bp)	136
Number of scaffolds (>1 kb)	107
Number of scaffolds (>50 kb)	71
Sequencing read coverage depth (fold)	196.72
GC-Content	53.04
No. of predicted protein-coding genes	14,721
Gene density (genes/Mb)	447.5
Average length of transcripts	1468.7
Average CDS length	1354.2
Average protein length	451.4
Average exon length	568.6
Average intron length	82.9
Spliced genes	9702 (66.0%)
Number of total introns	20,309
Median number of introns per gene	2.0
Number of total exons	35,010
Median number of exons per gene	2.0

**Table 2:** Genome annotation summary of *C. kikuchii* strain ARG_18_001.

Summary	Number
Number of protein-coding gene models	14,721
Number of models with BLAST hit	13,015 (88.4%)
Blast2GO annotation	6296 (42.8%)
PFAM annotation	5684 (38.6%)

**Table 3:** Summary of repetitive elements in the assembled genome of *C. kikuchii* strain ARG_18_001.

Summary	Number
Total of bases masked	178,815 (0.54%)
Number of simple repeats	3131
Number of low complexity repeats	358
Number of DNA transposons	68
Number of LTRs	2
Number of LINEs	254
Number of SINEs	21

## 2 Experimental design, materials, and methods

### 2.1 Genomic DNA extraction and sequencing

*Cercospora kikuchii* strain ARG_18_001 was isolated from a single conidium from soybean seeds of variety DM62R63 sampled that exhibited symptoms of purple seed stain during the 2018 harvest in San Pedro, Buenos Aires, Argentina. The isolation technique is described in [4]. This strain was deposited in the fungal culture collection of the Department of Plant Pathology, School of Agriculture, University of Buenos Aires (FAUBA, Argentina). Genomic DNA was isolated from hyphal tissue grown in potato dextrose broth for four days in darkness and constant agitation. The DNA extraction was carried out at the Institute of Microbiology and Agricultural Zoology (IMYZA -INTA) using a modified cetyltrimethylammonium bromide (CTAB) extraction protocol developed by [5]. Total DNA was quantified by fluorometry using a Picogreen dsDNA dye kit (Quant-iT, Invitrogen, by Life Technologies, CA, USA) with a Victor 3 plate reader.

Paired-end whole-genome shotgun libraries were constructed using the TruSeq Nano DNA (insert size 350 bp) library preparation kit following Illumina (San Diego, CA) protocols. Sequencing was performed using a NovaSeq 6000 sequencing system (Illumina) and yielded 65,202,278 reads.

### 2.2 Phylogenetic species identification

The isolate ARG_18_001 was identified by aligning seven nuclear loci (actin (*actA*), calmodulin (*cmdA*), nuclear ribosomal internal transcribed spacer region (*nrITS*), glyceraldehyde-3-phosphate dehydrogenase (*gapdh*), histone H3 (*his 3*), translation elongation factor 1-a (*tef1-alpha*) and beta tubulin (*tub2*)) with data from [6,7]. A maximum-likelihood phylogeny was then inferred in RAxML (Randomized Axelerated Maximum Likelihood) [8] assuming a GTRGAMMA model with *Septoria provencialis* CPC_12226 as an outgroup.

### 2.3 Genome assembly and annotation

Read trimming and filtering was performed using Trimmomatic [9] and merging of paired-end reads from shorter fragments was made using FLASH [10]. De novo assembly was carried out using the Celera Assembler [11] and then completed with Spades [12] using a wide range of k-mer values from 21 to 111 with a step of 2. The genome was annotated using FunGAP [13], tRNAscan-SE [14], rnammer [15] and MFannot (http://megasun.bch.umontreal.ca/cgi-bin/mfannot/mfannotInterface.pl) [16]. For predicting genes with FunGAP, the *C. kikuchii* ARG_18_001 genome assembly and the *C. beticola* 10.73.4 (Bioproject PRJNA294383) RNA-seq reads were used as inputs. To perform the functional annotation, we used hmmsearch [17] against PFAM database (v32.0) (e-value cut off ≤ 10e-5) and BLASTP [18] (e-value cut off ≤ 10e-10) against the NCBI nr database. To assign Gene Ontology [19] terms we used Blast2GO [20] and pfam2go table (http://www.geneontology.org/external2go/pfam2go) with the ragp R package (https://rdrr.io/github/missuse/ragp/). The repetitive regions, including tandem repeats and transposable elements, were detected using the repeat identification tool RepeatMasker [21].
